# Sudden cardiac death in the young: single-center study of Bari autopsy cases

**DOI:** 10.3389/fcvm.2025.1630511

**Published:** 2025-09-11

**Authors:** Cecilia Salzillo, Marco Matteo Ciccone, Francesco Introna, Vincenzo Ezio Santobuono, Biagio Solarino, Andrea Marzullo

**Affiliations:** ^1^ Pathology Unit, Department of Precision and Regenerative Medicine and Ionian Area, University of Bari “Aldo Moro”, Bari, Italy; ^2^ PhD Course in Public Health, Department of Experimental Medicine, University of Campania “Luigi Vanvitelli”, Naples, Italy; ^3^ Cardiology Unit, Interdisciplinary Department of Medicine, University of Bari “Aldo Moro”, Bari, Italy; ^4^ Legal Medicine Unit, Interdisciplinary Department of Medicine, University of Bari “Aldo Moro”, Bari, Italy

**Keywords:** sudden cardiac death, young, molecular autopsy, autopsy guidelines, epidemiology, prevention, genetic screening

## Abstract

**Introduction:**

Sudden Cardiac Death (SCD) is one of the main causes of death in the world, with a significant impact especially on young people. Sudden Cardiac Death in the Young (SCDY) is characterized by multifactorial etiology, which includes cardiomyopathies, myocarditis, channelopathies, aortopathies and coronary artery diseases. Despite progress in prevention, a significant percentage of these deaths remain unexplained without a thorough autopsy. This study aims to SCDY cases registered between 2016 and 2024, exploring the association between type of autopsy, age, sex, causes of death and temporal changes.

**Methods:**

Data relating to subjects who died for suspected SCDY, who underwent forensic, or hospital autopsy were retrospectively analysed. Investigations included type of autopsy (diagnostic or judicial), age (in years), sex, available clinical data, gross and histological findings, and cause of death. The data were divided by age groups (0–10, 11–20, 21–30, 31–40 years), sex and cause of death (arrhythmias, congenital heart defects, myocarditis, vascular dissections and cardiomyopathies). The temporal distribution of cases was also evaluated.

**Results:**

A total of 62 cases were analysed, with a prevalence of male subjects (70%). Forensic autopsies (65%) were more frequent than diagnostic findings (35%). The most represented age groups were 11–20 years (30%) and 21–30 years (25%). Unknown arrhythmias were the main cause (40%), followed by congenital heart disease (20%) and cardiomyopathy (15%). Congenital heart defects prevailed in newborns and children, while hypertrophic or arrhythmogenic cardiomyopathies were more frequently observed in young adults. Temporally, there has been a progressive increase in molecular autopsies and genetic diagnoses, in particular after the introduction of the AECVP (2017) and SCVP (2023) guidelines.

**Discussion:**

The findings highlight the need for a multidisciplinary approach to diagnosis of SCDY, with particular emphasis on molecular autopsy to identify genetic causes. The male predominance and age-related etiological differences underline the importance of specific preventive strategies, such as genetic screening in newborns and victims’ relatives. The increase in diagnoses over time reflects the effectiveness of updated guidelines, but it remains crucial to expand the mandatory nature of autopsies to improve understanding of the causes of SCDY and reduce the incidence of these tragic events.

## 1 Introduction

Sudden Death (SD) is a dramatic and unexpected event that can affect individuals of any age, frequently in those who appear to be in good health. It is generally defined as death occurring within one hour of symptom onset, or within 24 hours of the last time the individual was seen alive in unwitnessed cases, or in patients who are resuscitated following a cardiac arrest but subsequently die in hospital (1). In many cases, SD represents the first and only clinical manifestation of an undiagnosed underlying condition, making autopsy the sole means of determining the cause of death (2).

Among the causes of SD, Sudden Cardiac Death (SCD) holds a prominent role. It is defined as a natural and unexpected fatal event that occurs within one hour of symptom onset in an apparently healthy individual, or in someone whose known pathology was not expected to be life-threatening (3, 4). SCD represents a significant public health challenge: despite existing prevention strategies, it remains the leading cause of death worldwide (5).

Each year, approximately 350,000 SDs are estimated to occur in Europe, and between 300,000 and 400,000 in the United States, with an annual incidence of 1–2 per 1,000 inhabitants (6, 7) According to the European Society of Cardiology (ESC), incidence rates range from 36 to 128 deaths per 100,000 inhabitants annually (8). Among young individuals under the age of 30, the incidence of SCD is lower—approximately 1–2.8 per 100,000—but remains significant, with peaks noted between the ages of 14 and 21 (9, 10). In roughly one-third of cases, the cause of death cannot be determined, even following a comprehensive autopsy (11).

In Italy, the epidemiological assessment of SCD is hindered by the absence of a national registry. Available data rely primarily on ISTAT, with the sole exception of the Veneto Region, which has implemented its own regional surveillance system (3, 5). According to these sources, SCD accounts for approximately 50,000 deaths annually, while Sudden Cardiac Death in the Young (SCDY) affects around 1,000 individuals each year. In Veneto, the cumulative incidence of SCDY is estimated at 1 per 100,000 per year, with a higher incidence in athletes (2.3/100,000) compared to non-athletes (0.9/100,000) (3, 5).

SCDY, which affects individuals between the ages of 1 and 35 (12–14), is frequently attributed to hereditary or genetic causes, including cardiomyopathies, channelopathies, aortopathies, and coronary artery anomalies, although non-hereditary causes such as myocarditis may also be involved (5, 15–19). Among the most common conditions in the young are hypertrophic cardiomyopathy (36%) and arrhythmogenic right ventricular cardiomyopathy (20%). In older populations, ischaemic heart disease is predominant (75%), followed by dilated cardiomyopathies (15%) (13, 20).

In a significant proportion of cases, traditional autopsy alone is insufficient to identify the cause of death. In such instances, the Molecular Autopsy (MA)—a post-mortem genetic investigation—proves essential in detecting hereditary pathologies and initiating preventive screening for at risk family members (21–25).

**Figure 1:**
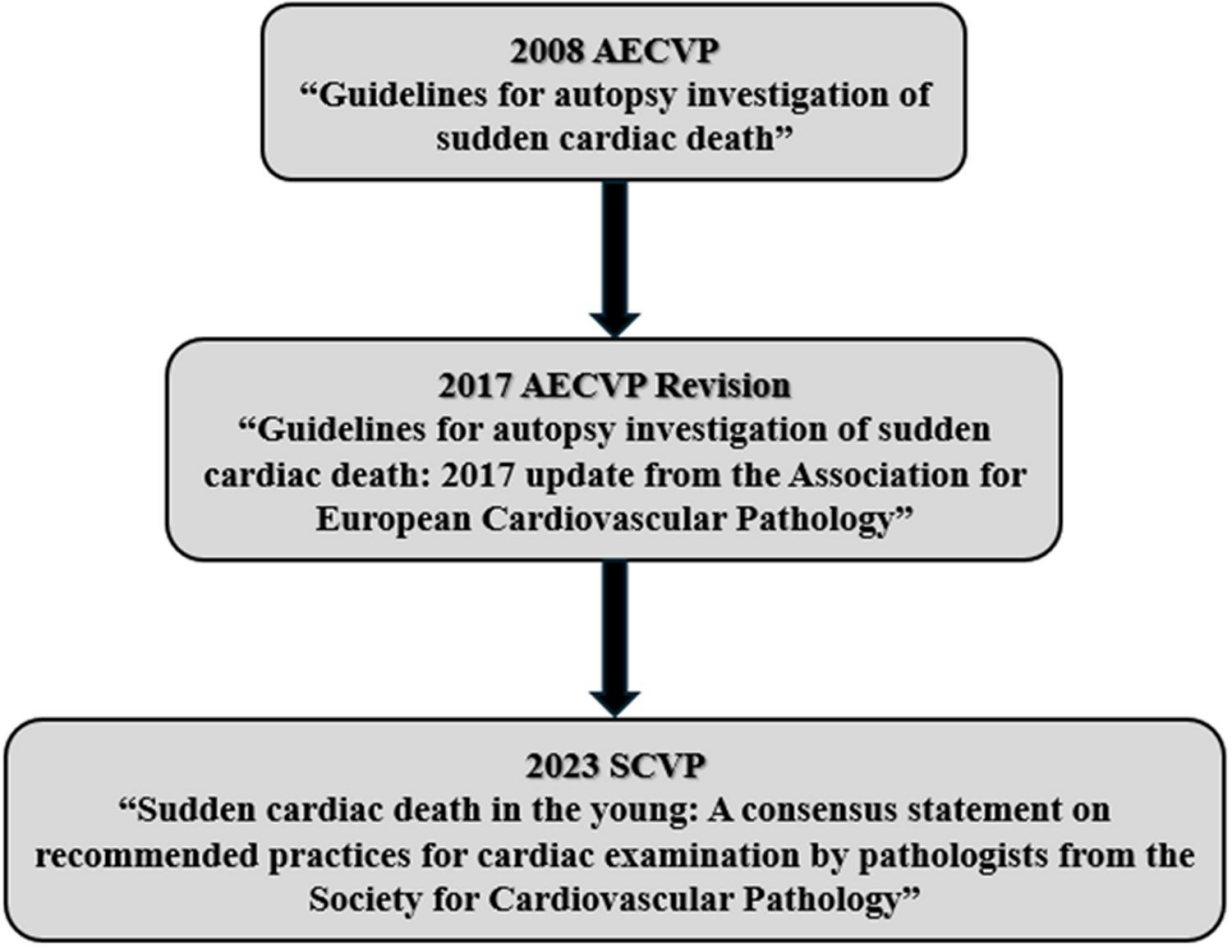
Time evolution of SCD guidelines and recommendations.

In response to the need for a standardised autopsy protocol, the European Association of Cardiovascular Pathology (AECVP) published the Guidelines for Autopsy Investigation of Sudden Cardiac Death in 2008. These guidelines represented a foundational effort to unify best practices across Europe (Figure 1). In 2017, the guidelines were revised to reflect advances in cardiovascular genetics and the importance of incorporating molecular testing and family medical history into the investigative process (Figure 1) (2).

In 2023, recognising the need to address younger populations specifically, the Society for Cardiovascular Pathology (SCVP) issued the consensus document Sudden Cardiac Death in the Young: A Consensus Statement on Recommended Practices for Cardiac Examination by Pathologists (Figure 1) (4). This publication reinforced the importance of post-mortem identification of genetic disorders and the pivotal role of autopsy in initiating life-saving diagnostic and therapeutic pathways for surviving relatives.

**Figure 2:**
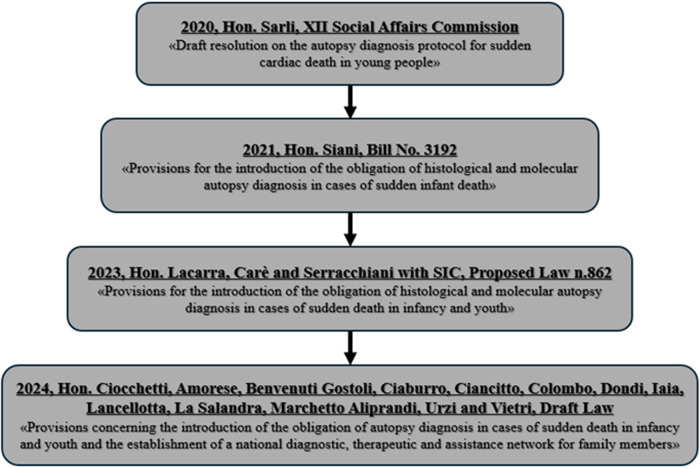
Legislative process in Italy: mandatory histological and molecular autopsy diagnosis for juvenile sudden death.

Growing awareness in Italy has led to multiple legislative proposals aimed at making histological and MA mandatory in all cases of juvenile SD (Figure 2). Since 2020, proposals have called not only for the obligatory diagnostic assessment but also for the development of a national network of reference centres, a national registry, structured diagnostic-therapeutic care pathways (PDTA), and wide-reaching prevention and information campaigns (5).

In this context, the autopsy—particularly in its most advanced and comprehensive form—should be viewed not merely as a diagnostic tool, but as the cornerstone of a secondary prevention strategy. Identifying a genetic condition in a deceased individual allows for the early identification and clinical management of at risk relatives, offering the potential to prevent future deaths. Thus, while SCD remains a profoundly tragic event, it may also serve as a critical catalyst for saving lives.

This study aims to investigate the autopsy diagnosis of cases SCDY, drawing on the most recent standards in the field—namely, the 2017 guidelines of the AECVP and the 2022 recommendations from the SCVP. By re-evaluating these cases in light of current diagnostic criteria, the study seeks not only to clarify the underlying causes of SCDY, but also to lay the groundwork for broader clinical and public health implications.

One of the central goals is to use the insights gained from MA to guide relatives of the deceased towards appropriate genetic counselling, clinical monitoring, and, when possible, preventive or therapeutic measures. Equally important is the effort to improve how information is communicated to families, ensuring that they are supported through a coordinated, multidisciplinary approach involving the cardio-pathologist, forensic pathologist, geneticist and cardiologist.

Lastly, by classifying and quantifying the causes of SCDY, the study aims to contribute to the establishment of a regional and national registry, helping to better understand the incidence of these conditions and ultimately improve strategies for early identification and prevention.

## 2 Materials and methods

### 2.1 Study design

This study was conducted through a retrospective analysis of autopsy reports from the Institutes of Pathology and Legal Medicine at the University of Bari “Aldo Moro”, in collaboration with cardio-pathologists and forensic pathologists.

### 2.2 Patient data source

Autopsy reports were sourced from the Unit of Pathology, Department of Precision and Regenerative Medicine and Ionian Area (DiMePRe-J), Bari Polyclinic. A digitised archive of histological and autopsy records covering the period from 2016 to 2024 was reviewed. The search was performed using relevant keywords including sudden cardiac death, sudden death, arrhythmia, myocardial infarction, dissection, aneurysm, myocarditis, fibrosis, fragmentation, and waviness.

Inclusion criteria comprised cases involving individuals aged ≤40 years.

Exclusion criteria included individuals older than 40 years, those with a confirmed non-cardiac cause of death, cases involving surgical correction of known congenital heart disease, trauma-related deaths, and drug-induced cardiac pathology.

### 2.3 Variables for analysis

Both diagnostic and judicial autopsies of individuals aged ≤40 years with suspected SCDY were included in the analysis. For each case, the following variables were collected: type of autopsy (diagnostic or judicial), age (in years), sex, available clinical data, gross and histological findings, and cause of death.

## 3 Results

The retrospective study analyzed cases of SCD in young subjects from 2016 to 2024 (Table 1), with the aim of better understanding the underlying causes, identifying possible correlations with hereditary pathologies and promoting prevention in blood relatives. Each year has highlighted peculiarities related to the characteristics of the cases analysed, the distribution of causes and the diagnostic implications.

**Table 1:** Autopsy cases from 2016 to 2024.

Year	Number	Autopsy	Age	Sex	Clinical	Macroscopic	Histology	Diagnosis death
2016	I433 (1)	FA	35	M	SD	N	FC	SCD: UA
2016	I3344 (2)	FA	27	M	SD	N	FC	SCD: UA
2016	I4927 (3)	FA	32	M	SD	N	FC	SCD: UA
2016	I5969 (4)	FA	36	M	SD	N	FC + CAIFT	SCD: UA
2016	I6609 (5)	FA	39	M	HC	N	IF + LHI + FC + CU	SCD: UA + MI
2016	I8127 (6)	FA	24	F	SD	N	SAA + RFEC + TMF	SCD: AD
2016	I10143 (7)	FA	32	M	SD	N	FC + CAIFT	SCD: UA
2016	I15040 (8)	FA	24	M	SD	N	AD + HI	SCD: AD
2016	I19356 (9)	FA	23	M	SD	N	FC	SCD: UA
2016	A29 (10)	HA	< 1	F	SD	N	FC + CU	SCD: SIDS + CA
2017	I5461 (1)	FA	29	M	SD	N	CN + IS + FC + CS	SCD: AMI
2017	I6975 (2)	FA	28	F	SD	N	TMF + FC	SCD: UA
2017	I12180 (3)	FA	14	M	SD	N	HC + CU	SCD: HC
2017	I13874 (4)	FA	19	F	SD	N	FC + CU	SCD: UA
2017	I16906 (5)	FA	40	M	SD	N	FC + CU + CS	SCD: UA
2017	A10 (6)	HA	<1	M	SD	RHP	HC	SCD: DC
2017	A45 (7)	HA	<1	M	SD	DIA + TGA	N	SCD: UA
2018	I80 (1)	FA	14	M	SD	N	LHI + CBN	SCD
2018	I19554 (2)	FA	11	M	SD + Joubert syndrome	N	FC	SCD: UA
2018	A27 (3)	HA	<1	M	SD	TGA + DIA + DIV	CN	SCD: CCHD
2019	I10361 (1)	FA	8	M	SD	N	LHI + FC + CU	SCD: MI
2019	A10 (2)	HA	<1	F	DIC	MCH	MCH	SCD: MCH
2020	I4125 (1)	FA	25	M	SD	N	FC + CU + LHI	SCD: UA
2020	I5128 (2)	FA	13	F	SD	N	CU	SCD: UA
2020	I9748 (3)	FA	38	M	SD	N	M + FC + CU	SCD: UA
2020	I12232 (4)	FA	17	M	SD: TAWT	N	CU	SCD: CC
2020	I12237 (5)	FA	18	M	SD: TAWT	N	FC	SCD: CC
2020	I15760 (6)	FA	36	M	SD	N	CAIFT + CU	SCD: UA
2020	I16587 (7)	FA	37	F	SD	N	CU + CAIFT + FI	SCD: UA
2020	I17889 (8)	FA	19	F	SD	N	CU + CAIFT	SCD: UA
2020	A31 (9)	HA	3	F	Hemolytic-uremic syndrome	EE	HI	SCD: CA
2020	A37 (10)	HA	<1	F	SD	CA + ASA	N	SCD: CCHD
2020	A38 (11)	HA	<1	F	SD	N	LHI + HCS	SCD: HMS
2021	I4484 (1)	FA	40	M	SD	N	CU + FC + HI + IF + HC + FI	SCD: UA
2021	I9602 (2)	FA	27	F	SD	N	CS + LHI + FC + HI	SCD: CT
2021	I11216 (3)	FA	3	F	SD	N	CU	SCD: UA
2021	I11766 (4)	FA	21	M	SD (private pool)	HC	CU + FC + CAIFT	SCD: UA
2021	I18030 (5)	FA	32	F	SD	N	FC	SCD: UA
2021	I21778 (6)	FA	15	M	SD (sports activity)	N	HC + MD + ISF	SCD: HC
2021	A19 (7)	HA	<1	M	SD	DIA + DIV	N	SCD: CCHD
2021	A32 (8)	HA	<1	F	SD	RVH + CAVC	EF + ISF	SCD: CCHD
2021	A37 (9)	HA	35	F	SD	RVH + MS	HC + FC + ISF	SCD: CCHD
2022	I11605 (1)	FA	38	F	SD	N	LHI + ISF	SCD: LM
2022	I14260 (2)	FA	1	F	SD	N	CU	SCD: UA
2022	I14300 (3)	FA	19	M	SD	DR: LEICA + LCIA	RFEC + TMF	SCD: MFD
2022	A33 (4)	HA	7	M	SD (Hodgkin's lymphoma)	CAT	CAT + LHI	SCD: KD
2022	A41 (5)	HA	17	M	SD (deceased at home + SD family history)	N	ISF + FC + CU	SCD: UA
2023	I302 (1)	FA	36	M	SD	N	CU	SCD: UA
2023	I304 (2)	FA	15	F	SD	N	CU + LHI	SCD: UA
2023	I5489 (3)	FA	40	M	SD	N	NI (microabscesses) + CN + CU	SCD: HS
2023	I5802 (4)	FA	30	M	SD	N	FC + M	SCD: UA
2023	I17885 (5)	FA	29	M	SD	N	CU + CAIFT	SCD: UA
2023	I23008 (6)	FA	24	F	SD	N	EF + ISF + CU	SCD: UA
2023	A07 (7)	HA	0	F	SD	DIA (suspected Trisomy 21)	N	SCD: CCHD
2023	A10 (8)	HA	<1	M	SD	DIA + CAVC	N	SCD: CCHD
2023	A18 (9)	HA	23	M	SD	MB (complete) LAD	MB (complete) LAD	SCD: MB (SCN5A)
2024	I177 (1)	FA	23	M	SD (deceased at home)	N	CU	SCD: UA
2024	I586 (2)	FA	26	F	SD: TAWT	N	CU	SCD: CC
2024	I5283 (3)	FA	27	M	SD (private pool)	HC	EF + ISF + HC + CU	SCD: HC
2024	I10897 (4)	FA	38	M	SD (quarrel)	N	HC + CU + CAIFT	SCD: CC
2024	I23790 (5)	FA	35	M	SD	N	IS + CU	SCD: CS
2024	A14 (6)	HA	<1	F	SD (parvovirus B19 positive)	N	LHI	SCD: LM

Autopsy: FA, forensic autopsy; HA, hospital autopsy. Sex: M, male; F, female. Clinical: SD, sudden death; HC, hypertrophic cardiopathy; DIC, disseminated intravascular coagulation; TAWT, traffic accident without trauma. macroscopic: N, negative; RHP, right heart predominant; DIA, atrial septal defect; TGA, transposition of the great arteries; DIV, ventricular septal defect; MCH, massive cardiac hemorrhage; EE, endocardial ecchymosis; CA, coarctation of the aorta; ASA, aberrant subclavian artery; HC, hypertrophic cardiopathy; RVH, right ventricular hypertrophy; CAVC, complete atrioventricular canal; MS, mitral stenosis; DR, dissection and rupture; LEICA, left extracranial internal carotid artery; LCIA, left common iliac artery; CAT, coronary aneurysm-thrombosis; MB, myocardial bridge; LAD, left descending artery. Histology: FC, fragmentation of cardiomyocytes; CAIFT, coronary artery intimal fibrous thickening; IF, intramural fibrosis; LHI, lympho-histiocytic inflammation; CU, cardiomyocyte undulation; SAA, structural alteration aorta; RFEC, rarefaction and fragmentation of the elastic component; TMF, tunica media fibrosis; AD, aortic dissection; HI, hemorrhagic infiltration; N, negative; CN, necrosi coagulativa; IS, ischemic scar; CS, coronary stenosis; HC, hypertrophic cardiomyocytes; CBN, contraction band necrosis; MCH, massive cardiac hemorrhage; M, myocardiosclerosis; FI, fat infiltration; MD, myocardial disarray; EF, endocardial fibrosis; HCS, hydropic cardiomyocyte swelling; ISF, interstitial fibrosis; CAT, coronary aneurysm-thrombosis; NI, neutrophilic inflammation; MB, myocardial bridge; LAD, left descending artery. diagnosis death: SCD, sudden cardiac death; UA, unknown arrhythmia; MI, myocardial inflammation; AD, aortic dissection; CA, conduction anomalies; AMI, acute myocardial ischemia; HC, hypertrophic cardiomyopathy; DC, dilated cardiomyopathy; CCHD, complex congenital heart disease; MCH, massive cardiac hemorrhage; CC, commotio cordis; CA, cardiocirculatory arrest; HMS, hypoxic myocardial suffering; CT, cardiac tamponade; LM, lymphocytic myocarditis; MFD, muscular fibrodysplasia; KD, Kawasaki disease; HS, hemorrhagic shock; MB, myocardial bridge; CS, coronary stenosis.

In 2016 (Table 1), ten cases were recovered, highlighting a prevalence of SD attributable to unknown arrhythmic causes in six out of ten cases. These cases were histologically characterized by fragmentation and waviness of the myocardial fibers, a morphological finding compatible with an arrhythmogenic substrate. Two cases were attributed to aortic dissection, a rare condition in young people, which suggests the need to consider underlying vascular pathologies as possible risk factors. Finally, the case of a newborn girl who died from SIDS associated with an anomaly of the cardiac conduction tissue highlights the importance of studying the conduction tissue in sudden infant deaths.

In 2017 (Table 1), the cases reviewed showed greater etiological diversity. Hypertrophic cardiomyopathy was identified in an adolescent, a condition that is a major cause of SCDY athletes. Myocardial infarction was found in a young adult with critical coronary stenosis, highlighting the importance of also monitoring traditional cardiovascular risk factors in young people. Finally, two cases of congenital heart disease in newborns confirmed the relevant role of structural abnormalities in the pathogenesis of SCD.

The year 2018 (Table 1) sees the emergence of rare and complex conditions, such as Joubert syndrome, associated with arrhythmic death. This case highlights the link between genetic syndromes and cardiac dysfunction. Furthermore, the observation of fibrosis and necrosis in young subjects reinforces the idea that inflammatory and ischemic processes can play a crucial role even at an early age.

In 2019 (Table 1), the two cases analysed highlighted the centrality of myocarditis as a cause of SCDY. In particular, an acute myocarditis led to the death of an eight-year-old child, suggesting that acute cardiac inflammation, often undiagnosed during life, represents an underestimated danger.

In 2020 (Table 1), a significant increase in cases was recorded, eleven in total, many of which were attributable to arrhythmogenic causes. Two cases of commotio cordis, which occurred in contexts of non-evident trauma, underline the importance of always considering this possibility in the presence of otherwise unexplained deaths. Fragmentation and undulation of myocardial fibers are, however, a recurrent histological sign, consolidating their value as indicators of arrhythmic death.

In 2021 (Table 1), nine cases showed an increase in cardiomyopathies, particularly hypertrophic cardiomyopathies, often detected in young athletes. These results reinforce the importance of cardiovascular screening programs in athletes to prevent fatal events. Furthermore, deaths in patients with systemic diseases suggest that general health conditions may amplify cardiac risk.

In 2022 (Table 1), unusual causes emerged, such as an arterial dissection attributable to muscular fibrodysplasia and a death from coronary thrombosis related to Kawasaki disease. These cases highlight the need for multidisciplinary diagnostic approaches to identify rare vascular and inflammatory conditions.

The 2023 results (Table 1) highlighted the importance of genetic testing, with one case associated with an SCN5A pathogenic variant and myocardial bridging, both known to increase the risk of arrhythmias. These results suggest that targeted genetic testing may be essential to identify at risk family members and prevent the eventual death.

In 2024 (Table 1), the six cases analysed reaffirmed the diversity of underlying causes, including critical coronary stenosis, commotio cordis, and myocarditis. A case of a female newborn positive for parvovirus B19 highlighted the role of viral infections in SCD, underlining the need for paediatric surveillance.

The results clearly show the etiological variability of SCDY, with a variety of causes ranging from genetic anomalies and congenital heart disease to inflammatory diseases. The integration of multidisciplinary approaches and the implementation of genetic and cardiac screening programs could represent fundamental strategies to reduce the incidence of these tragic events.

## 4 Discussion

The study analyses cases of SCDY collected between 2016 and 2024. The approach adopted allowed to explore changes over time, the correlation between demographic variables such as age and sex, the type of autopsy, the causes of death and the social and clinical context of each event. This discussion integrates the results in the light of the most recent guidelines and scientific recommendations, emphasizing the importance of autopsy diagnosis for public health (2, 4).

The distinction between judicial autopsy and diagnostic finding is emblematic of the challenges in detecting and classifying SCD.

Judicial autopsies represented most of the cases studied, reflecting the urgency of clarifying the causes of SD in non-clinical contexts, often of a suspicious or violent nature. This type of autopsy focuses on analyses that go beyond the macro and microscopic evaluations typical of the anatomo-pathological approach and include toxicological assessments.

However, diagnostic feedback, although less frequent, has an equally critical role, especially in hospital and paediatric settings. It is also essential in the forensic field, to understand deaths associated with congenital heart disease, channelopathies or infections, often underestimated during life. The poor adoption of diagnostic feedback in some population groups underlines the urgency of implementing the mandatory nature of these procedures, as suggested by the recent Proposal for a Law (5).

**Figure 3:**
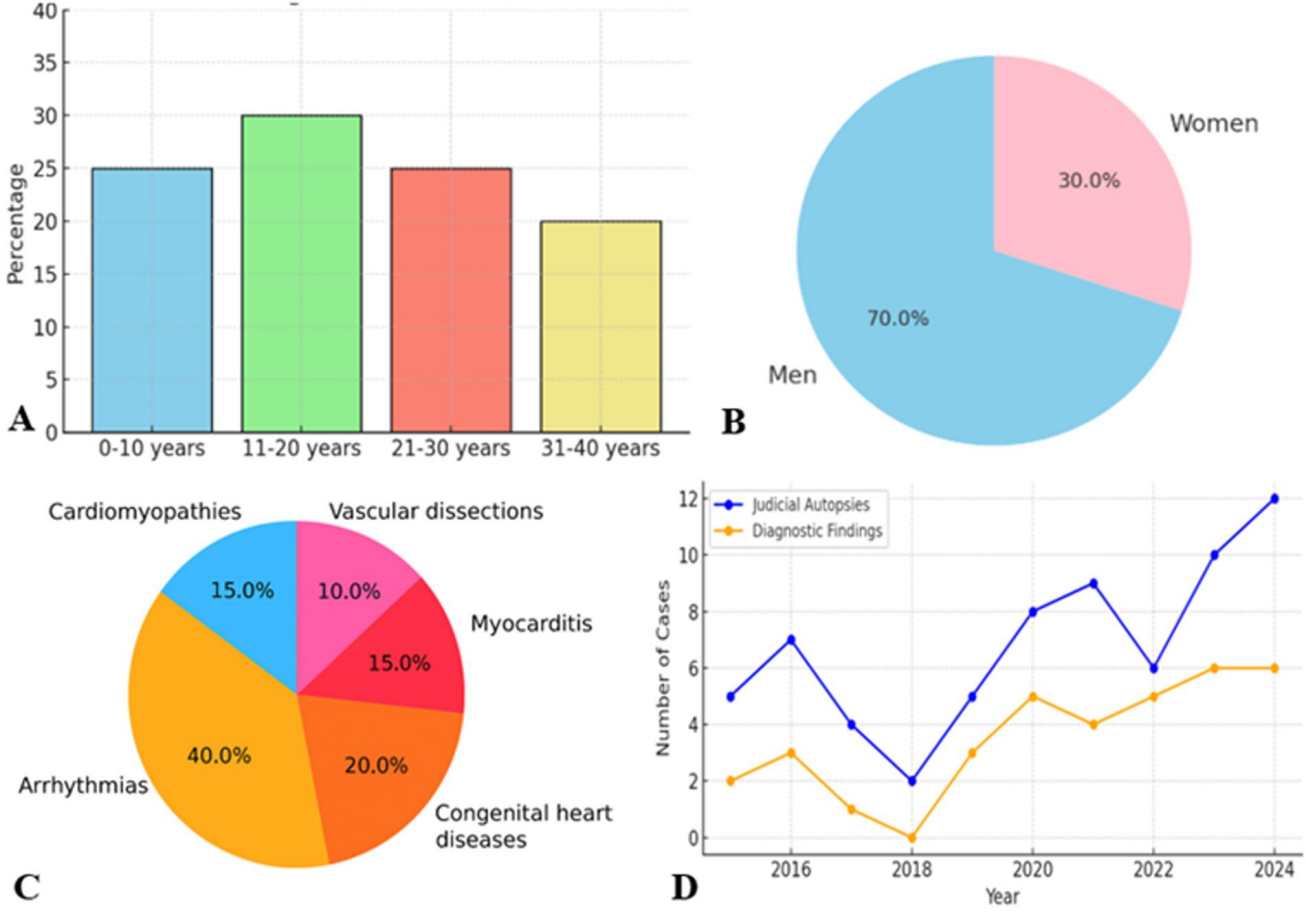
**(A)** Age distribution. **(B)** Gender distribution. **(C)** Distribution death causes. **(D)** Autopsy time distribution.

The results reveal a clear etiological gradient related to age (Figure 3A).

In neonates and children under 10 years of age, congenital heart disease is the leading cause of SD. This includes structural defects such as ventricular septal defect, coarctation of the aorta, and coronary artery anomalies. Many cases remain undiagnosed during life, underscoring the need for more effective neonatal screening and early genetic testing.

In young adults aged 20–40 years, arrhythmic causes predominate, often related to hypertrophic or arrhythmogenic cardiomyopathies, or cardiac channelopathies. This trend reflects not only the pathophysiological dynamics, but also the diagnostic limitations in pre-mortem settings. In the cases analysed, hypertrophic cardiomyopathy was a recurrent cause.

Another key element is the marked prevalence of male cases (Figure 3B), in line with epidemiological evidence.

Men are at higher risk of SCD than women, probably due to a combination of hormonal, genetic and behavioural factors. Androgens are associated with increased susceptibility to ventricular arrhythmias. However, the gender distribution is more balanced in neonates and children, suggesting that at an early age the risk is more closely linked to genetic or congenital factors than to sex.

The identified causes of death reflect a highly variable panorama (Figure 3C).

**Figure 4:**
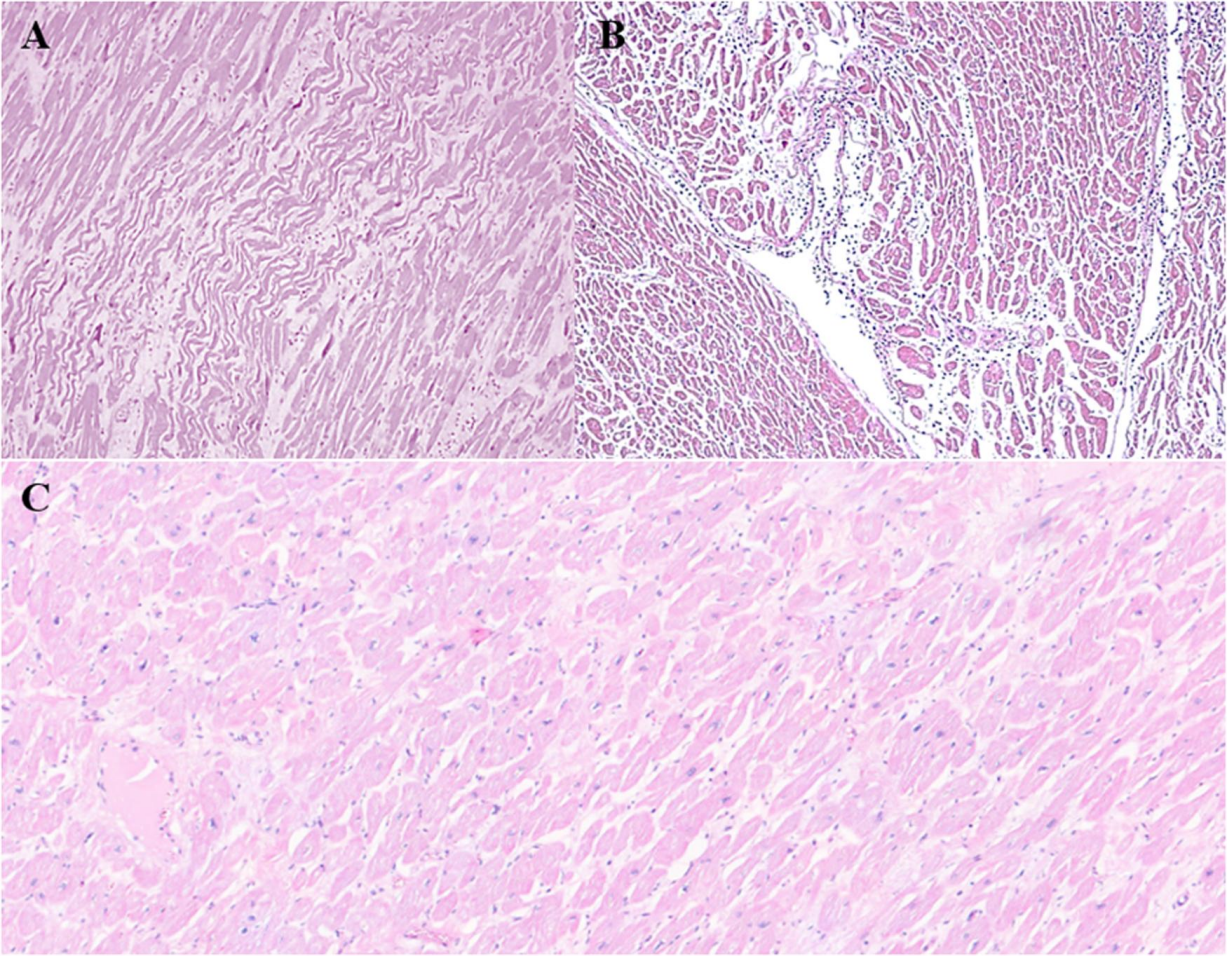
**(A)** Diffuse waviness and fragmentation of myocardial fibers (HE, 10×). **(B)** Focal nonspecific inflammatory infiltrate (HE, 10×). **(C)** Hypertrophic-regressive aspects of cardiomyocytes and interstitial fibrosis (HE, 20×).

Arrhythmias of unknown origin are the predominant cause in all age groups. These cases, characterized by nonspecific histological signs such as fragmentation and waviness of myocardial fibers (Figure 4A), require the use of advanced diagnostic techniques, such as MA to identify significant genetic mutations associated with channelopathies and cardiomyopathy.

Genetic phenotyping in SCDY cases is essential to understand the cause of death and to guide preventive strategies in at-risk family members. Recent studies have highlighted how the main nosological categories underlying SCDY are represented by hereditary cardiomyopathies and channelopathies, both caused by pathogenic variants with autosomal dominant transmission, with highly heterogeneous penetrance and phenotypic variability (21, 23).

The most frequently pathogenic variants in hypertrophic cardiomyopathy are MYBPC3 and MYH7, which together account for approximately 70% of cases. Specifically, mutations in the MYBPC3 gene are found in 40%–45% of patients, while those in the MYH7 gene are found in 15%–25%. In arrhythmogenic cardiomyopathy, the genes most commonly involved are PKP2, mutated in 10%–45% of cases, followed by DSP in 10%–15%, DSG2 in 10%, and, more rarely, JUP and DSC2, which present mutations in 1%–2% of cases. In dilated cardiomyopathy, however, the most frequently mutated gene is TTN, present in 20%–25% of patients. This is followed by LMNA, with mutations in 8% of cases, RBM20 in 1%–5%, and SCN5A, whose alterations are found in 2%–3% of cases (5).

Regarding channelopathies, among the most frequently involved genes, SCN5A represents a key gene and is involved in both long QT syndrome type 3 and Brugada syndrome and has been identified in over 20%–25% of SCDY cases with a structurally normal heart but is also responsible for dilated cardiomyopathy.Furthermore, pathogenic variants in KCNQ1 and KCNH2 are responsible for LQT1 and LQT2 forms, RYR2 is implicated in catecholaminergic polymorphic ventricular syndrome, KCNQ1, KCNJ2 and KCNH2 related to short QT syndrome (5).

Variable penetrance and the frequent absence of an obvious clinical phenotype in carriers make it essential to correlate genetic, histological, and clinical/anamnestic data. The integration of MA, through panels including next-generation sequencing (NGS), whole-exome sequencing (WES), and the identification of copy number variants (CNVs), today allows not only to formulate a post-mortem diagnosis in subjects who have died suddenly, but also to offer targeted and personalized screening to family members (26, 27).

This supports the use of electrocardiogram screening and a genetic panel for the recognition of high-risk pathogenic variants such as SCN5A, KCNQ1, KCNH2, RYR2, KCNQ1, KCNJ2, KCNH2, MYBPC3, MYH7, PKP2, DSP, DSG2, JUP, DSC2, TTN, LMNA, RBM20, and targeted follow-up, including possible prophylactic implantable cardiac defibrillator, for primary prevention in asymptomatic carriers (2, 21).

**Figure 5:**
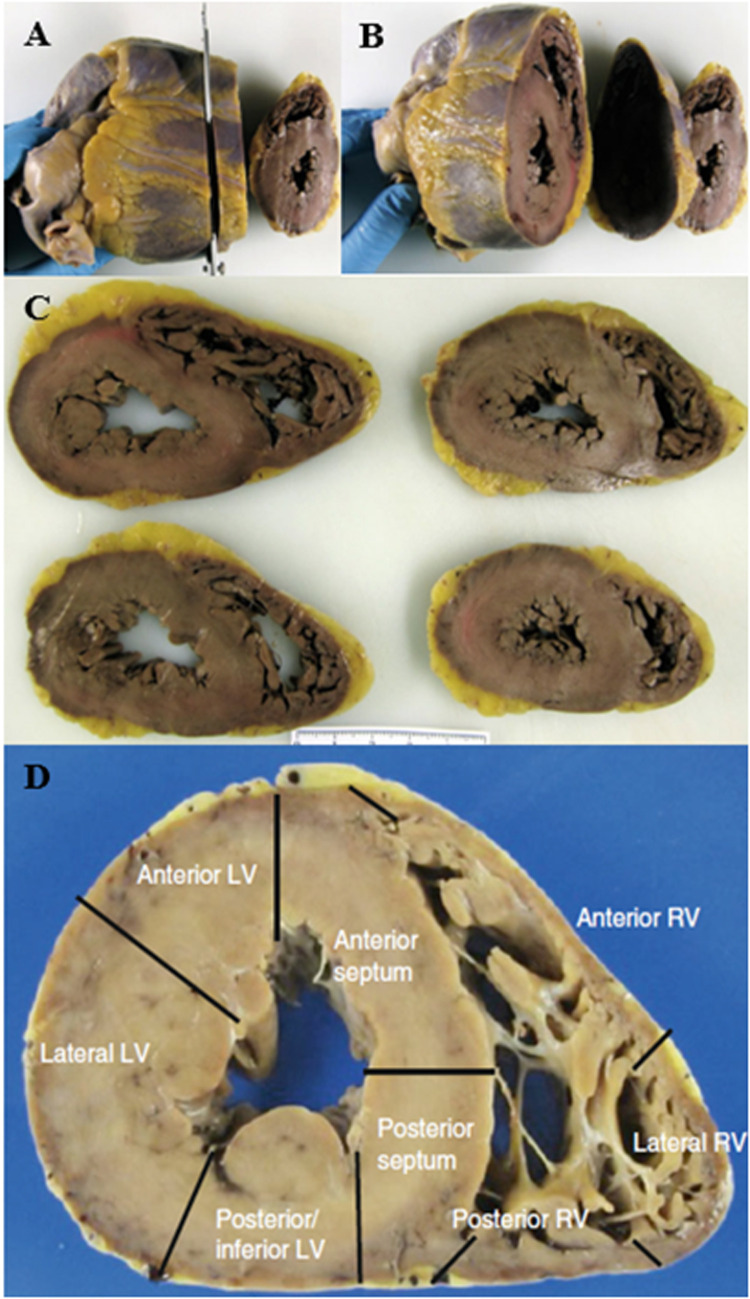
**(A–C)** Protocol to study the heart by transverse sectioning from the apex to the papillary muscles. **(D)** Mapping of a transverse section of the heart. (37).

From a histological point of view, the evaluation of myocardial fiber fragmentation is a very critical point since it is difficult to define whether it is true and pathological or artefactual depending on the sampling mapping of the heart. Specifically, the critical sites are the interventricular septum and the posterior wall, where the myocardial fibers of the right and left ventricle intersect, mimicking these histological findings. Furthermore, fragmentation can also be caused by technical artifact due to vibration of the microtome during cutting, which is generally localized and not diffuse. Therefore, the sampling technique according to apico-subvalvular transverse sections is essential, placing the heart on the section table in a progressive direction and the complete sampling of a section according to mapping (Figure 5).

**Figure 6:**
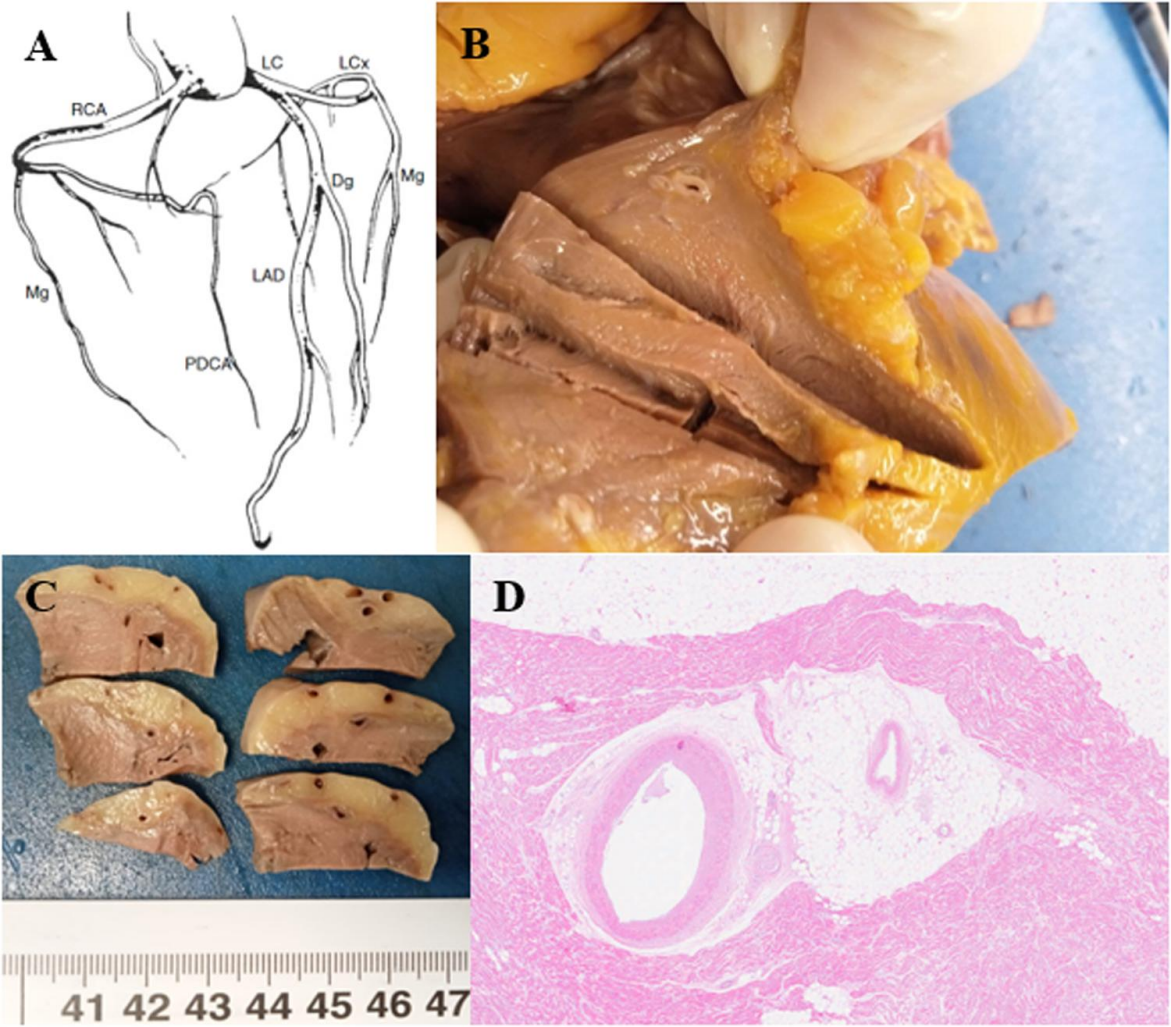
**(A)** Coronary tree diagram. RCA right coronary artery, PDCA posterior descending coronary artery, LC trunk left coronary artery, LAD left anterior descending coronary artery, Dg diagonal, LCx left circumflex, Mg marginal (37). **(B,C)** The coronary arteries are patent with the presence of a complete myocardial bridge of the anterior descending artery extending for 3 cm and 3.5 cm from the emergence of the coronary artery. **(D)** The anterior descending coronary artery is characterized by intramyocardial tunneling as a “complete myocardial bridge” and with a discrete fibrous thickening of the intima (HE, 1.25×).

Congenital heart disease is common in newborns and children and is often caused by hereditary and congenital pathologies (17). Therefore, the lack of diagnosis of these conditions during life highlights the importance of more in-depth prenatal and paediatric surveillance. In these, congenital anomalies of the coronary arteries must also be considered, among which the major cause of death is myocardial bridge characterized by a “tunnelized” coronary artery. It is therefore essential to study the coronary tree by making transverse sections at a distance of 0.5 cm, starting from the origin of the coronary arteries and following their course (Figure 6).

Myocarditis is a major cause of death in young adults and presents a histological diagnostic challenge both in the living and at autopsy. In fact, the Dallas criteria (28), introduced in 1987, define myocarditis as the presence of an inflammatory infiltrate in the myocardium accompanied by necrosis or degeneration of cardiomyocytes, excluding an ischemic origin, but are not applicable to autopsy cases because they were originally defined on endomyocardial biopsy.

An additional element of complexity in the histological diagnosis lies in the variety of inflammatory patterns observed in myocarditis. Lymphocytic, eosinophilic, neutrophilic and giant cell forms present different microscopic features, each of which may be associated with specific aetiologies, including viral infections, autoimmune reactions and drug hypersensitivity. Lymphocytic myocarditis, the most common subtype, is often related to viral infections, although it is not always possible to identify a specific pathogen. This particularly insidious form can evolve in clinical silence, representing a significant risk for fatal events (29).

The association between myocarditis and SCD is particularly relevant in young adults (30) and is considered one of the main non-ischemic causes of death. It is estimated that up to 6%–10% of SCD cases in Italy are attributable to myocarditis, although these numbers may be underestimated. Autopsy studies have revealed that, in cases of SD, the presence of subclinical or focal myocarditis is often found in the absence of a previous clinical diagnosis. This highlights the importance of a thorough post-mortem histological examination with extensive sampling, which often reveals unexpected inflammatory infiltrates associated with myocardial damage and a focal distribution of the inflammatory infiltrate (Figure 4B).

Vascular dissections are rare but lethal, often associated with genetic syndromes such as Marfan syndrome, Loeys-Dietz syndrome, Ehlers-Danlos vascular syndrome and muscular fibrodysplasia. In these conditions, histological differential diagnosis is difficult because they share some histopathological features, in particular fragmentation and/or loss of elastic fibers, but at present it is not yet possible to differentiate them based on particular morphological patterns; therefore, the association with clinical and genetic features is essential for the diagnosis.

Cardiomyopathies are a major cause of SCDY. Histological and macroscopic diagnosis of postmortem cardiomyopathies is particularly complex due to various factors involving both the natural changes that occur in tissues after death and the difficulties related to the identification of the hallmarks of cardiac pathologies.

After death, the heart undergoes changes due to autolysis and decomposition, which can make it difficult to observe microscopic details such as muscle cell hypertrophy or fibrosis (Figure 4C), typical of many cardiomyopathies.

**Figure 7:**
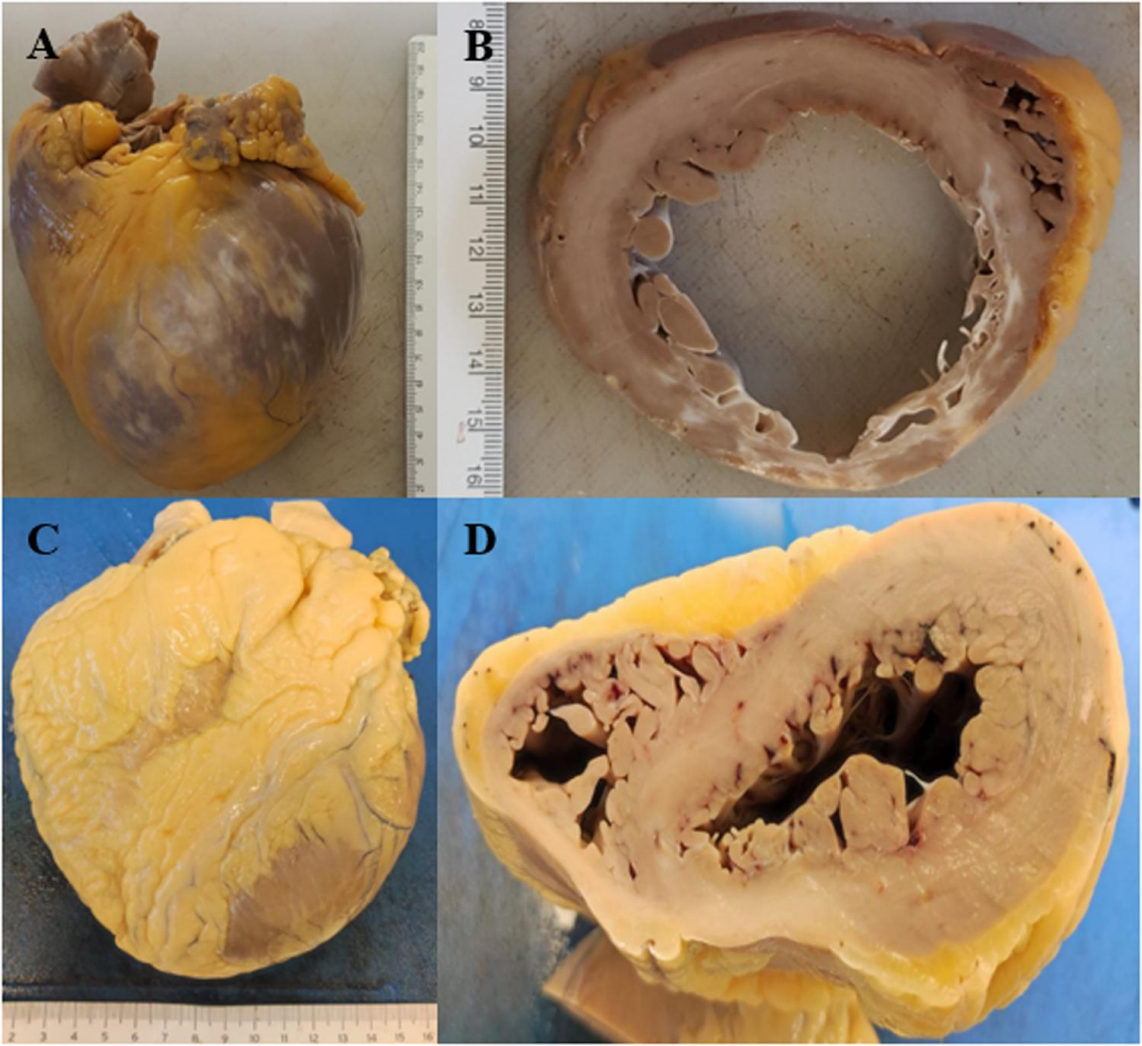
**(A)** Dilated cardiomyopathy. **(B)** Cross-section of dilated cardiomyopathy with diffuse whitish areas. **(C)** Hypertrophic cardiomyopathy. **(D)** Cross-section of hypertrophic cardiomyopathy.

Furthermore, many cardiomyopathies present similar macroscopic signs, such as cardiac enlargement or ventricular dilation (Figure 7), which can easily be confused with other pathologies, such as ischemic heart disease or alterations due to hypertension.

Microscopic analysis is also problematic, since phenomena such as fibrosis or inflammatory infiltrates are not exclusive to a given condition but are also found in other cardiac diseases. The difficulties increase when the sample taken does not come from the most representative areas of the heart, such as the interventricular septum, or when the tissues are not well preserved.

In addition, postmortem diagnosis lacks the support of clinical information, such as the patient's medical history or the results of advanced diagnostic tests, which in life help to guide the diagnosis. In many cases, cardiomyopathies also have a genetic component, which cannot be detected by traditional macroscopic or histological methods and requires MA.

Longitudinal analysis reveals significant changes over time (Figure 3D), linked to the evolution of diagnostic techniques and the introduction of updated guidelines.

Before 2017, many diagnoses were based on basic macroscopic and histological signs. However, with the 2017 AECVP guidelines update, a standardized approach was introduced, which significantly improved the ability to identify less obvious causes of death, such as channelopathies and genetic mutations.

Another turning point occurred with the 2023 SCVP recommendations, which emphasized the importance of MA and multidisciplinary collaboration. These developments contributed to a greater understanding of the causes of SCD, also leading to an increase in genetic diagnoses and the creation of diagnostic-therapeutic pathways for the families of victims.

The study clearly shows the crucial importance of autopsies, both forensic and diagnostic, to understand the causes of SD. The differences observed based on the type of autopsy, age, sex and causes of death highlight the need for a personalized and multidisciplinary approach.

It is essential to implement policies that make histological and MA mandatory in cases of SD, as proposed by the most recent legislation. At the same time, it is essential to promote preventive genetic and cardiac screening, especially in risk groups, such as newborns, children with congenital anomalies and young athletes. Only an integrated approach can help reduce the incidence of these deaths and improve public health.

Furthermore, the results of our case series are consistent with those reported by international studies conducted in different socio-geographical contexts.

An Indian study (31) reported a negative autopsy rate of 35.6%, comparable to the 40% observed in our study. This data underscores the crucial role of genetically based arrhythmias among the causes of SCDY not attributable to structural abnormalities.

A British study (32) confirmed that sudden arrhythmic death in subjects with structurally normal hearts represents the majority of SCD cases in athletes, at 78%, with a diagnostic yield of 17% using MA, which focused predominantly on genes associated with cardiomyopathies rather than channelopathies.

A Chinese study (24) has highlighted how a significant proportion of cases with structurally normal hearts are associated with mutations in genes affecting intercellular junctions, ion channels, or the sarcomere, reinforcing the importance of systematically integrating genetic analysis into postmortem investigations.

Another significant contribution comes from a Danish study (33) conducted nationwide over a 10-year period, which showed a significant reduction in the crude incidence of SCDY, adjusted for age and sex. Although the study did not show significant changes in the distribution of the main causes of SCDY, it suggests a decreasing trend in incidence over time, in line with what has also been observed in US and Swedish studies. In particular, the most marked reduction was observed in women, although the difference between the sexes was not statistically significant.

An important element that emerges from our case history is represented by the high prevalence of cases classified as unknown arrhythmias (40%), in which no macroscopic structural anomalies or currently recognized genetic alterations emerged. This data, consistent with what has been reported in other studies, highlights the diagnostic limitations still present in conventional post-mortem investigations and underlines the existence of arrhythmic mechanisms not yet fully understood or identifiable with current techniques. This category could include forms of channelopathies not yet characterized epigenetic and environmental alterations and genetic variants of unknown significance (VUS) that escape current diagnostic capacity (34, 35).

Although VUS cannot be considered pathogenic in the absence of functional or familial evidence, they raise important questions in terms of clinical interpretation, family management, and preventive decisions. The presence of VUS contributes to diagnostic uncertainty and represents one of the main challenges in the transition of forensic genetics into clinical practice (35).

It is therefore essential to promote the development of internationally shared databases (36) and familial co-segregation studies to refine the classification of the detected variants.

Overall, these international data reinforce the observations emerging from our case series and underline the value of forensic genetics in improving diagnostic accuracy and familial prevention of SCDY, in an evolving epidemiological context.

## 5 Limitation

Our study has several limitations that should be considered when interpreting the results.

Although it represents one of the largest national series of SCDY cases, including 62 autopsy-confirmed subjects, the sample size still remains relatively small, particularly given the diversity of underlying diagnoses, which may limit both the statistical power and the generalisability of the findings.

The retrospective, single-centre design of the study introduces the potential for selection bias and may limit the applicability of the results to other clinical or geographical settings.

We acknowledge that retrospective case selection based on keywords in digital registries represents a potential source of methodological bias. Specifically, this strategy can generate both inclusion and exclusion bias. On the one hand, actually relevant cases may be omitted due to imprecise or non-standardized clinical-pathological coding, and on the other, irrelevant cases may be incorrectly included due to the presence of generic or ambiguous terms. The lack of a uniform diagnostic vocabulary, especially in free-language autopsy reports, can compromise the sensitivity and specificity of the selection process. Therefore, although this approach was necessary in the retrospective data collection phase, we recognize the need, in future prospective studies, to implement standardized case classification and tracking criteria, as well as the adoption of controlled clinical dictionaries to reduce semantic error and improve the reliability of the collected data.

A further significant limitation is that clinical and genetic evaluation of family members was not always feasible or conducted in a systematic manner, thereby restricting the analysis of inheritance patterns.

In addition, the clinical heterogeneity of the cases poses a challenge in defining specific risk profiles and hampers meaningful comparison between subgroups.

Furthermore, data related to COVID-19 infection or vaccination status were not systematically recorded in our retrospective database. However, we intend to integrate these variables in future prospective collections to evaluate the possible association between viral infections, myocardial inflammatory response and SCDY.

These factors must all be considered when interpreting the findings and drawing conclusions from the study.

## 6 Proposed operational protocol for the establishment of a regional-national registry on SCDY

Considering the increasing focus on the prevention of SCDY, the establishment of a centralised regional-national registry has been proposed to enable the systematic collection and analysis of cases occurring in individuals aged 40 years or younger. The registry serves a dual purpose: firstly, to support epidemiological research into SCDY, facilitating the identification of risk factors and the development of targeted prevention strategies; secondly, to offer clinical, genetic, and psychological support to at-risk families.

The operational protocol outlined applies to all healthcare institutions, forensic pathology departments, and laboratories involved in managing these cases, and encourages collaboration with local, regional, and national authorities. Oversight of the registry is entrusted to a Registry Coordination Committee (CCR), which is responsible for general supervision, ensuring compliance with data protection regulations, and maintaining data quality. At the territorial level, data collection and management are coordinated by Regional Referents (RRs), working in synergy with Local Operating Units (UOLs), which verify and transmit the data. Cardiovascular pathologists and forensic pathologists provide autopsy and histopathological findings, while geneticists and cardiologists contribute genetic analyses and conduct familial screenings, with a particular focus on the electrophysiological evaluation of cardiac function.

**Figure 8:**
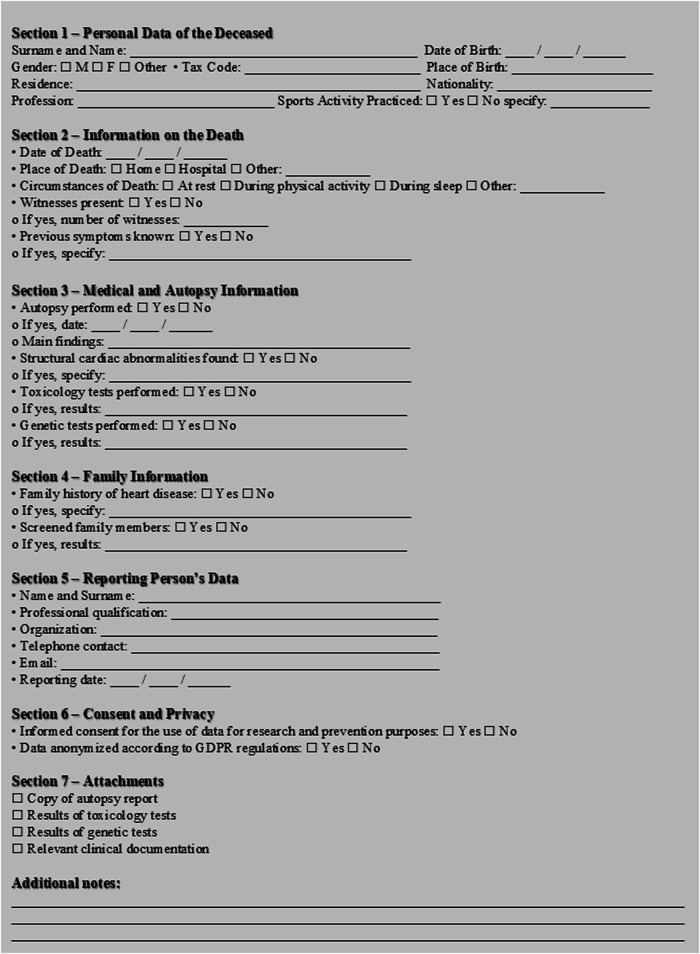
SCDY registry reporting form. *Instructions for sending the form: Sending method: The completed form must be sent within 15 days of death to the Regional/National Registry SCDY: Email or Secure web platform. • Accepted format: digitally completed PDF or scanned paper form.

The system is underpinned by a secure electronic database, accessible via a web-based platform, and supported by standardised data collection forms—both in paper (Figure 8) and digital format. Epidemiological and statistical analyses are conducted using dedicated software tools.

**Figure 9:**
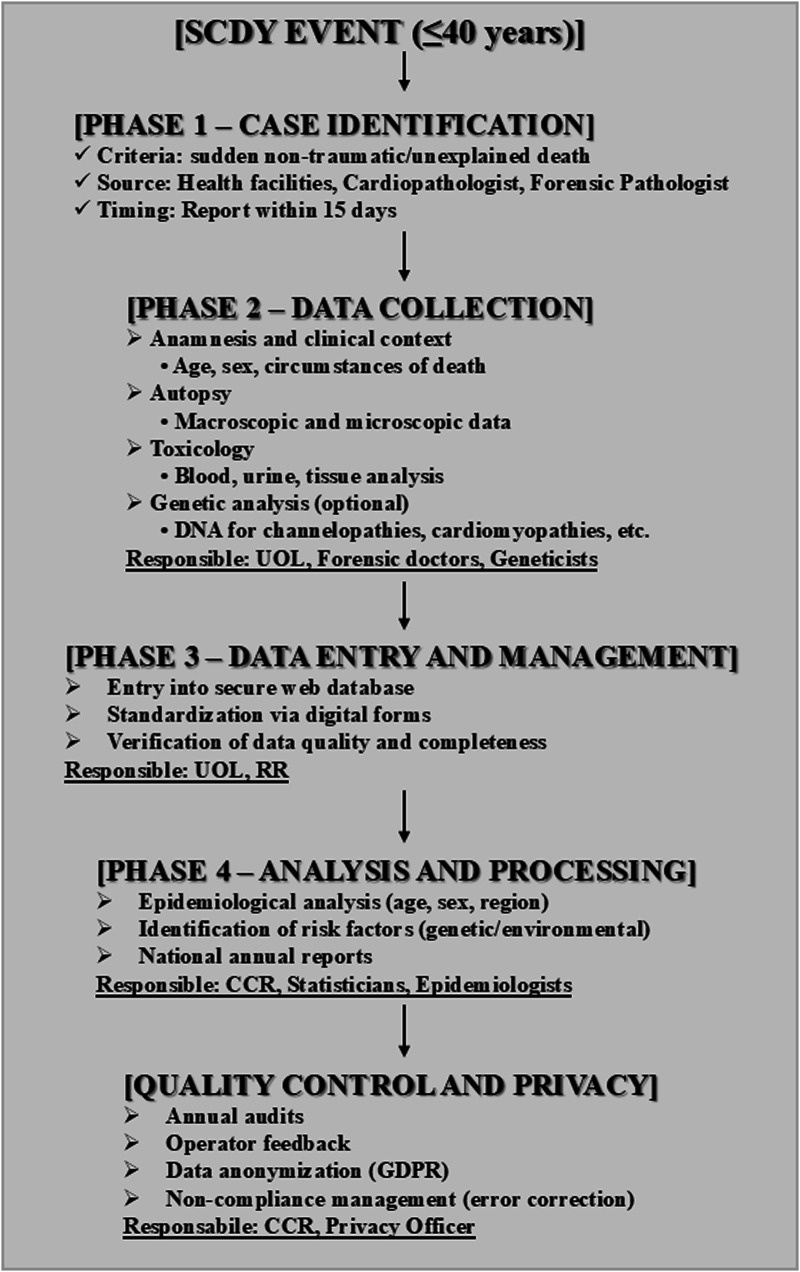
SCDY registry scientific diagram. *CCR, registry coordination committee, RR, regional representative, UOL, local operating units.

The protocol is structured into four phases (Figure 9). The first involves the identification of cases, specifically sudden, non-traumatic, or unexplained deaths in individuals aged ≤40 years, confirmed as cardiac in origin through autopsy or ancillary testing. Healthcare facilities, pathologists, and forensic doctors are required to report each case within 15 days. In the second phase, comprehensive data collection is undertaken, covering personal and circumstantial information, autopsy and toxicology results, and, where available, genetic testing for mutations associated with channelopathies, cardiomyopathies, and other heritable conditions. The third phase entails the entry of data into the electronic system, followed by regular quality control checks to ensure completeness and accuracy. In the fourth phase, data is processed to generate epidemiological insights, identify risk factors, and produce annual reports summarising key findings.

The protocol includes specific monitoring and evaluation mechanisms, such as annual audits and feedback collection from participating personnel. Stringent adherence to current data protection regulations (GDPR) is ensured, with complete anonymisation of data prior to its use for research or analytical purposes. In cases of non-compliance, corrective measures are in place to address any deficiencies in data handling procedures.

**Figure 10:**
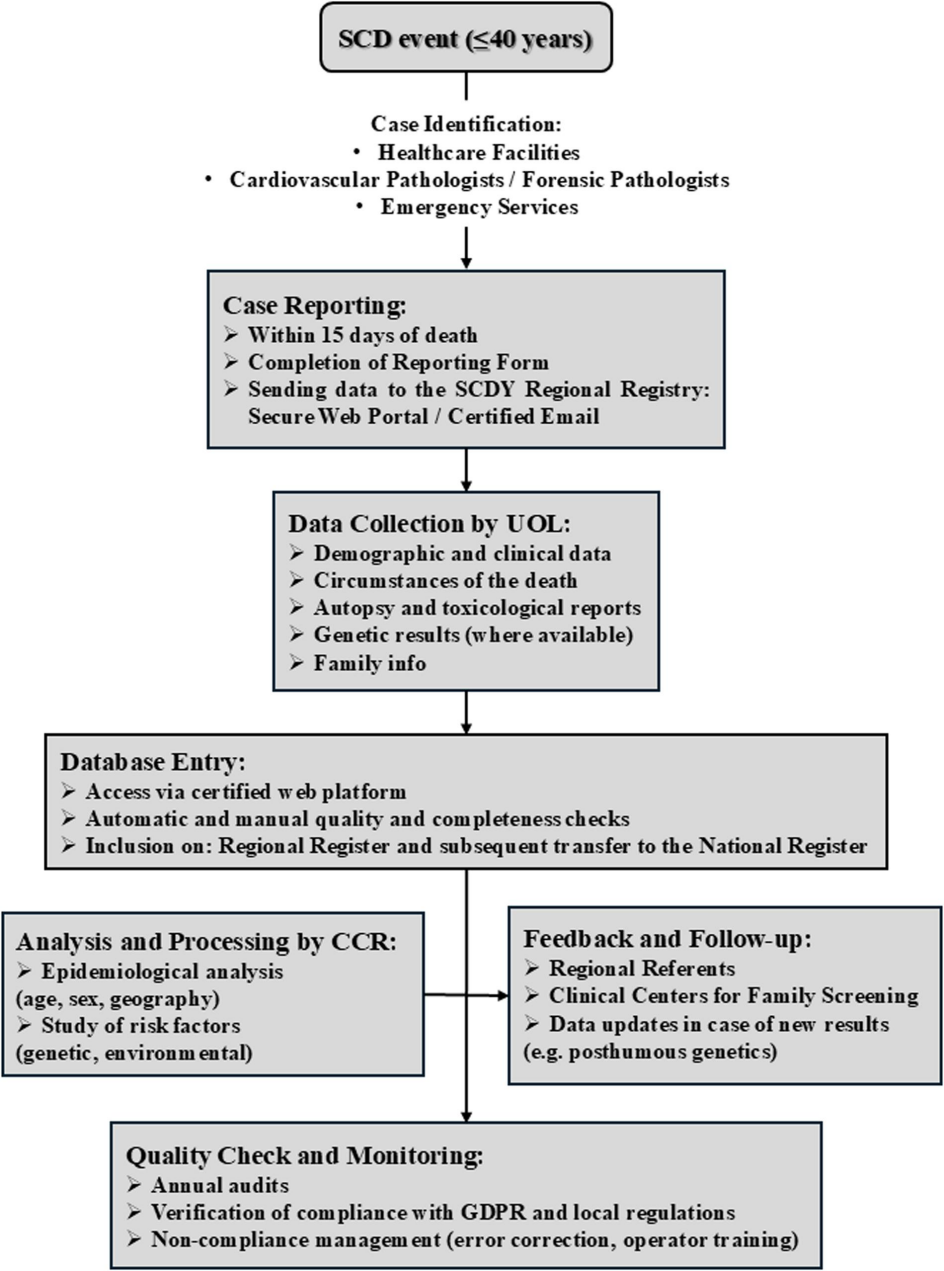
SCDY register data flow operational scheme. *UOL, local operating units; CCR, registry coordination committee.

Developed in accordance with international guidelines (AECVP, SCVP) and national regulations governing health registries, the protocol is accompanied by the relevant case-reporting forms, genetic data handling guidelines, and a diagram outlining the operational workflow (Figure 10). Through its structured and collaborative approach, this registry aims to enhance understanding of major inherited cardiac conditions and contribute meaningfully to the reduction of fatal events among young individuals.

## 7 Conclusions and future perspectives

This retrospective study has provided deeper insight into the causes and characteristics of the pathologies responsible for SCD, with a particular focus on younger populations. The results underscore the critical importance of employing advanced diagnostic techniques, such as MA, to determine the cause of death in cases that are often difficult to interpret. This approach not only offers clear answers to bereaved families but also serves as a vital tool for preventive care, enabling the identification of potential genetic or molecular risks among relatives.

Another key issue emerging from this study is the pressing need for uniform and shared guidelines to standardise investigation protocols in cases of SD. Such harmonisation is essential to ensure accurate diagnoses and to collect valuable data at both national and international levels, ultimately fostering a better understanding of the underlying pathologies.

Looking ahead, it is evident that investment on multiple fronts is required. Firstly, the establishment of regional and national registries dedicated to these conditions could be transformative, offering essential epidemiological insights into the incidence and characteristics of SD. Simultaneously, the training and continuous professional development of healthcare professionals must be prioritised, ensuring that they remain up to date with the most advanced diagnostic techniques and are able of recognising the signs of potentially fatal conditions.

Particularly noteworthy is the role of the cardiovascular pathologist, a specialist in pathology with specific, preferably certified, training in the recognition and definition of cardiac and vascular diseases. This level of expertise is essential for identifying subtle morphological findings that might otherwise be overlooked during a generalist evaluation. Failing to recognise such findings can result in missed opportunities for clinical and genetic evaluation of family members, crucial steps for both public health and preventive medicine.

Equally important is the engagement of the scientific and legislative communities in advocating for the mandatory performance of autopsy in cases of SCDY. Such a measure would not only enhance prevention efforts but also help build a robust support network for affected families by offering access to targeted diagnostic and therapeutic pathways.

The systematic adoption of MA with targeted post-mortem genetic testing represents a paradigm shift in the management of SCDY cases. Early identification of high-risk pathogenic variants allows for the activation of a preventive clinical pathway in family members with a positive test, up to the indication for subcutaneous defibrillator implantation in asymptomatic subjects.

Moreover, it is essential to continue supporting scientific research aimed at the development of new preventive and therapeutic strategies. Only through close collaboration between research, clinical practice, and health policy can we hope to mitigate the societal impact of these conditions and provide meaningful responses to those affected by such tragedies.

While the path forward is undoubtedly complex, the findings of this study represent a significant step towards a more personalised and prevention-oriented approach to medicine. The ultimate challenge for modern healthcare is to integrate MA as a routine component of post-mortem practice, ensuring accurate genetic interpretation of identified variants and clinically actionable outcomes for asymptomatic individuals.

To achieve this, it is mandatory to establish multidisciplinary teams comprising cardiovascular pathologists, forensic pathologists, geneticists, cardiologists, paediatricians, and general practitioners. Only through such coordinated collaboration can we ensure comprehensive care, informed family screening, and meaningful preventive action.

## Data Availability

The original contributions presented in the study are included in the article/Supplementary Material, further inquiries can be directed to the corresponding author.
